# Comparison of cardiovascular risk factors between children and adolescents with classes III and IV obesity: findings from the APV cohort

**DOI:** 10.1038/s41366-021-00773-x

**Published:** 2021-04-07

**Authors:** Thomas Reinehr, Sascha R. Tittel, Rolf Holle, Susanna Wiegand, Ines Gellhaus, Johannes Hebebrand, Susanne Greber-Platzer, Christian Denzer, Sabine Linke, Wieland Kiess, Reinhard W. Holl

**Affiliations:** 1 Department of Pediatric Endocrinology, Diabetes and Nutrition Medicine, Vestische Hospital for Children and Adolescents Datteln, University of Witten/Herdecke, Witten, Germany; 2 German Center for Diabetes Research, Institute of Epidemiology and Medical Biometry, University of Ulm, Ulm, Germany; 3 Institute for Medical Informatics, Biometry and Epidemiology, University Hospital, Ludwig-Maximilians-University Munich, Munich, Germany; 4 Charité–Universitätsmedizin Berlin, corporate member of Freie Universität Berlin, Humboldt-Universität zu Berlin, and Berlin Institute of Health, Berlin, Germany; 5 Consensus Group Obesity Training for Children and Young Adults, Rehabilitation Clinic for Children and Adolescents, Sylt, Germany; 6 Department of Child and Adolescent Psychiatry, Psychosomatics and Psychotherapy, University Hospital Essen, University of Duisburg—Essen, Essen, Germany; 7 Comprehensive Center Pediatrics, Department of Pediatrics and Adolescent Medicine, Division of Pediatric Pulmonology, Allergology and Endocrinology, Medical University Vienna, Vienna, Austria; 8 Department of Pediatrics and Adolescent Medicine, Division of Pediatric Endocrinology and Diabetes, University Medical Center Ulm, Ulm, Germany; 9 Katholisches Kinderkrankenhaus WILHELMSTIFT gGmbH, Children’s Hospital, Hamburg, Germany; 10 University of Leipzig, Hospital for Children and Adolescents, Leipzig, Germany

**Keywords:** Obesity, Epidemiology

## Abstract

**Objective:**

Obesity is associated with many cardiovascular risk factors (CVRF) in childhood. There is an ongoing discussion whether there is a linear relationship between degree of overweight and deterioration of CVRFs justifying body mass index (BMI) cut-offs for treatment decisions.

**Methods:**

We studied the impact of BMI-SDS on blood pressure, lipids, and glucose metabolism in 76,660 children (aged 5–25 years) subdivided in five groups: overweight (BMI-SDS 1.3 to <1.8), obesity class I (BMI-SDS 1.8 to <2.3), class II (BMI-SDS 2.3–2.8), class III (BMI-SDS > 2.8–3.3), and class IV (BMI-SDS > 3.3). Analyses were stratified by age and sex.

**Results:**

We found a relationship between BMI-SDS and blood pressure, triglycerides, HDL cholesterol, liver enzymes, and the triglycerides–HDL-cholesterol ratio at any age and sex. Many of these associations lost significance when comparing children with obesity classes III and IV: In females < 14 years and males < 12 years triglycerides and glucose parameters did not differ significantly between classes IV and III obesity. Prevalence of dyslipidemia was significantly higher in class IV compared to class III obesity only in females ≥ 14 years and males ≥ 12 years but not in younger children. In girls < 14 years and in boys of any age, the prevalences of type 2 diabetes mellitus did not differ between classes III and IV obesity.

**Conclusions:**

Since a BMI above the highest BMI cut-off was not associated consistently with dyslipidemia and disturbed glucose metabolism in every age group both in boys and girls, measurements of CVRFs instead of BMI cut-off seem preferable to guide different treatment approaches in obesity such as medications or bariatric surgery.

## Introduction

Obesity is associated with many cardiovascular risk factors (CVRF) such as hypertension, dyslipidemia, and impaired glucose metabolism in childhood [1, 2]. These CVRFs are expected to lead to premature death later in life. It has been demonstrated in a large longitudinal study over 40 years that mortality due to cardiovascular events rose with increase of body mass index (BMI) in adolescents [3]. Many studies reported a significant relationship between degree of overweight and CVRFs in children and adolescents [4–8]. This relationship is mediated by sex and age since CVRFs deteriorated during puberty and improved at the end of puberty [9].

There is an ongoing discussion whether a linear dose-response relationship of increasing degree of overweight on the presence of CVRFs [3] plateaus above a certain threshold. It has been demonstrated in one previous study that children and adolescents with class III obesity as defined by 140–160% of the 95th percentile of their BMI did not differ in their CVRFs from children and adolescents with class IV obesity as defined by >160% of the 95th percentile of their BMI [10]. This finding may have practical relevance, because indications for different treatment approaches for obesity such as medications or bariatric surgery are usually based on defined cut-off points of degree of overweight [11, 12]. However, large confirmatory studies are still lacking.

Therefore, we studied the impact of degree of overweight on CVRFs in a large cohort of children and adolescents with special focus on classes III and IV obesity subgroups using different classifications of obesity. By stratifying the analyses by sex and age groups, we tried to identify possible influencing factors on this relationship. We hypothesize according to the study of Zabarsky et al. [10] that children and adolescents with class IV obesity do not differ in their CVRFs from children and adolescents with class III obesity.

## Subjects and methods

Based on the German guidelines for diagnostics and treatment of overweight children and adolescents [13], a computer software based on the visual foxpro 9.0 compiler was developed for standardized prospective documentation of overweight children and adolescents (APV) in 1999 (www.a-p-v.de) [14]. Participation in this quality control program is the precondition for accreditation of obesity treatment centers by the German Obesity Society (DAG) and for funding of the treatment by health insurances. Anthropometric parameters, metabolic control, and treatment (modality and intensity of lifestyle interventions) are documented longitudinally by the software. The software allows standardized patient reports, local aggregation of data, and patient selection according to multiple criteria. Anonymized data are transmitted for central analysis. Each participating center complies with its local ethical and data management guidelines. Inconsistent data are reported back to the centers twice a year for correction.

A total of 224 centers specialized in pediatric obesity care in Germany, Austria, and Switzerland participated in this quality assessment. All patients at the age of ≥5 years and up to 25 years presenting at these institutions with a BMI > 25 kg/m^2^ or BMI-SDS > 1.3 in the years 2006–2016 were included in the analysis. Children with syndromes were excluded. In the longitudinal analyses, children and adolescents with bariatric surgery and children and adolescents with long-term inpatient treatment > 6 months were also excluded.

German population-based reference data for BMI were used as recommended by the International Task Force of Obesity (IOTF) [15]. The degree of overweight was quantified using Cole’s Box Cox-transformation, which normalizes the BMI skewed distribution in childhood and expresses BMI as a standard deviation score (SDS-BMI) [16]. Degree of overweight was categorized in five classes based on two different approaches: (a) based on BMI-SDS as suggested by IOTF [overweight: BMI-SDS 1.3 to <1.8, class I obesity (mildly obese): BMI-SDS 1.8 to <2.3 corresponding to 97th to <99.5th BMI percentile, class II obesity (moderately obese): BMI-SDS 2.3–2.8 corresponding to 99.5th to <99.9th BMI percentile, class III obesity (extremely obese): BMI-SDS >2.8–3.3 [15], class IV obesity (super obese): BMI-SDS >3.3] and (b) as suggested by Zabarsky et al. [10] overweight as 85th–95th percentile of BMI and obesity according to the 95th percentile of BMI (obesity class I: 100–120% of 95th percentile of BMI, class II: >120–140% of the 95th percentile of BMI, class III: >140–160% of the 95th percentile of BMI, class IV: >160% of the 95th percentile of BMI) [10]. Class I obesity corresponds to a BMI 30–35 kg/m^2^ in adults; class II to a BMI 35–40 kg/m^2^ in adults and class III to a BMI > 40 kg/m^2^ in adults.

To account for a potential impact of puberty on CVRFs, we divided our study population in three classes for boys and girls, respectively: boys aged < 12 years assuming prepuberty, boys aged 12 to <16 years assuming puberty, and boys aged ≥ 16 years assuming postpuberty; girls aged < 10 years assuming prepuberty, girls aged 10 to <14 years assuming puberty, and girls aged ≥ 14 years assuming postpuberty.

To adjust for differences among different laboratory methods, the multiple of the mean method was applied to mathematically standardize HbA1c measurements to the Diabetes Control and Complications Trial reference range (4.05–6.05%; 20.7–42.6 mmol/mol) [17]. An oral glucose tolerance test (oGTT) was performed according to recent international guidelines [18].

Non-HDL cholesterol was calculated by subtracting HDL cholesterol from total cholesterol. Dyslipidemia was defined by total cholesterol levels > 200 mg/dl or HDL < 35 mg/dl or LDL > 130 mg/dl or triglycerides > 150 mg/dl according to German guidelines [19]. The triglycerides to HDL-cholesterol ratio (T/H) was used as an indirect measurement of insulin resistance. Arterial hypertension was defined by blood pressure above the 95th percentile or >140/90 mmHg using German percentiles [20, 21] and diabetes by fasting glucose ≥ 126 mg/dl or 2-h glucose in oGTT ≥ 200 mg/dl or HbA1c > 6.5% [18].

The CVRFs, transaminases (aspartate aminotransferase (AST), alanine aminotransferase (ALT), glutamate-pyruvate transaminase (γGT)), and the prevalence of hypertension, dyslipidemia, and type 2 diabetes mellitus (T2DM) were compared between the patients with classes III and IV obesity. Analyses were performed stratified by sex and age.

### Statistics

Statistical evaluation was performed by SAS-version 9.4 (SAS Inst. Inc., Cary, NC, USA). Data are presented as median values and interquartile range. The relationship between BMI-SDS and CVRFs was calculated by linear regression models. Group comparisons were performed using Wilcoxon’s rank sum test for continuous outcomes and the chi-squared test for dichotomous outcomes within each of the four strata. Changes in BMI-SDS during follow-up were also calculated via linear regression and adjusted for age, sex, migration background, and lifestyle intervention. Two-sided *p* values were adjusted for multiple testing by Bonferroni stepdown method and *p* < 0.05 was considered significant.

## Results

**Fig. 1:**
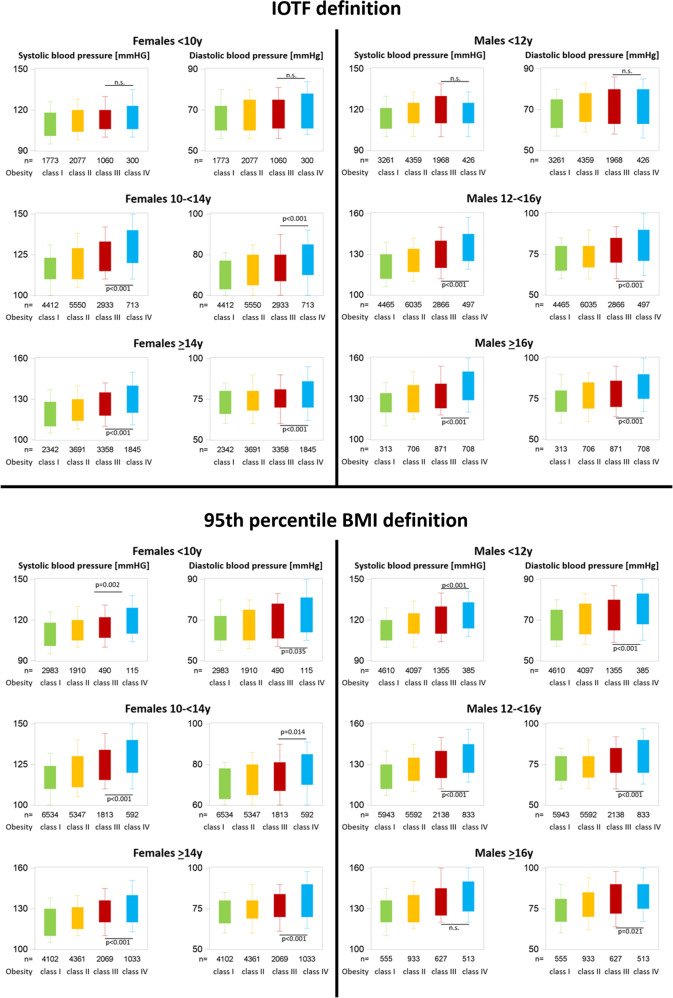
Blood pressure stratified by obesity class, sex, and age using two different obesity class definitions.

**Fig. 2:**
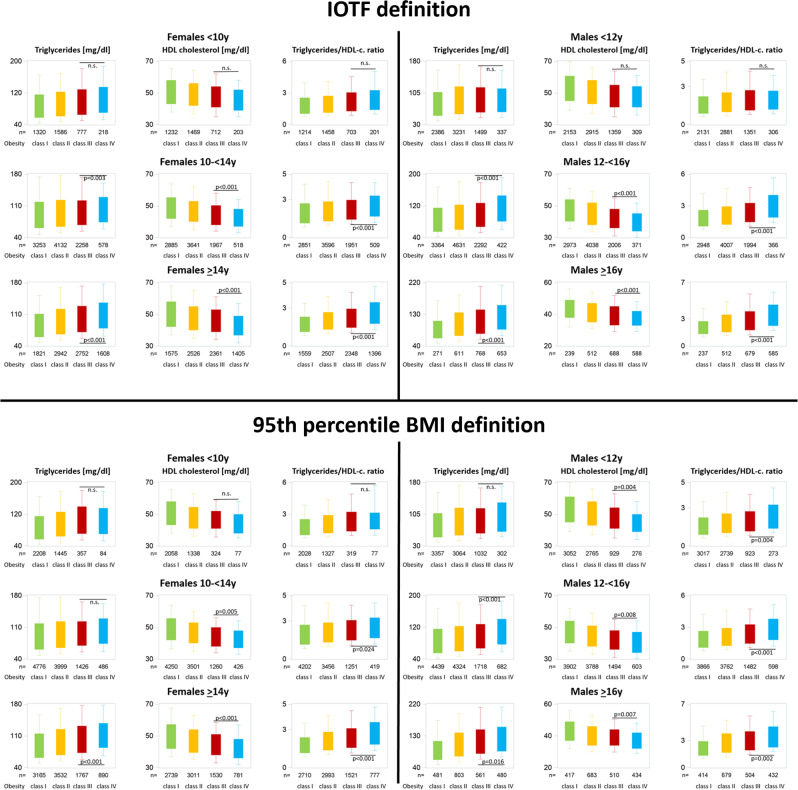
Lipids stratified by obesity class, sex, and age using two different obesity class definitions.

**Fig. 3:**
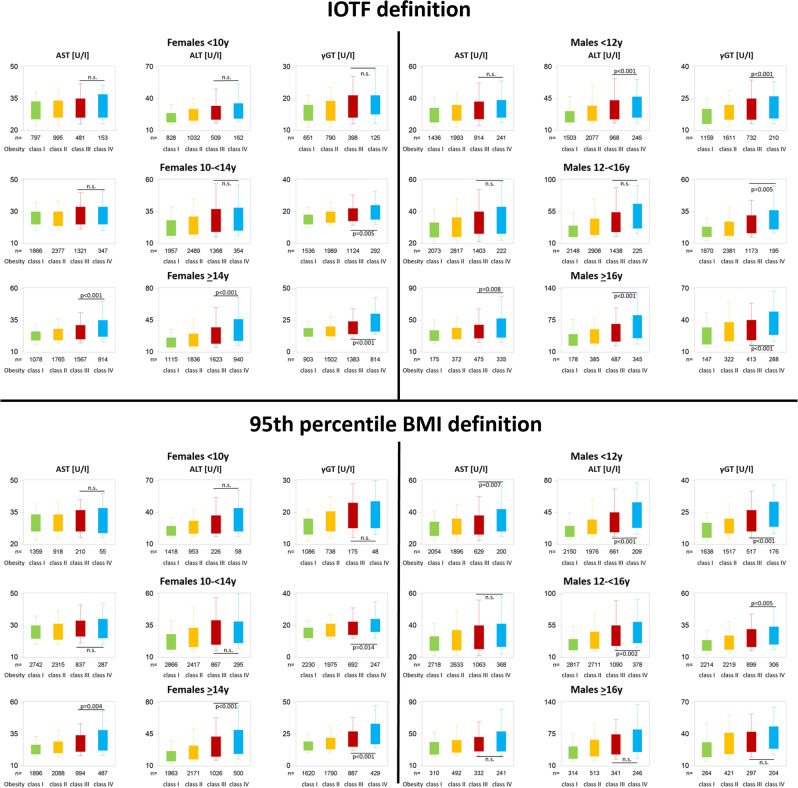
Liver enzymes stratified by obesity class, sex, and age using two different obesity class definitions.

**Fig. 4:**
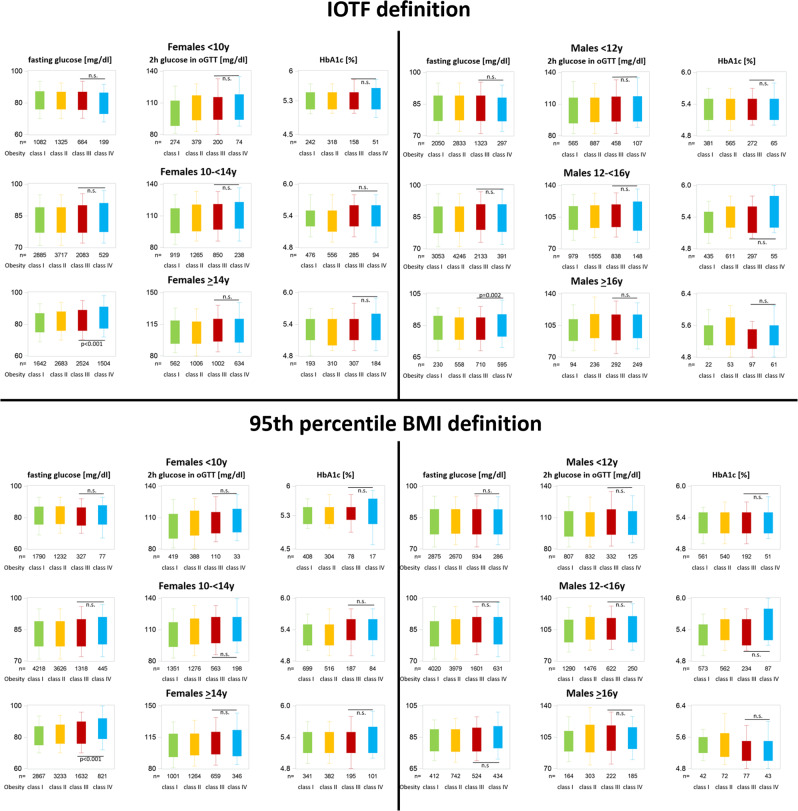
Glucose metabolism parameters stratified by obesity class, sex, and age using two different obesity class definitions.

A total of 76,660 children and adolescents met the inclusion criteria. The baseline characteristics are presented in Table 1. The CVRFs are demonstrated in Figs. 1–4 stratified by age, sex, and respective definition of obesity classes.

**Table 1:** Characteristics of the study cohort.

	BMI-SDS					BMI percentile				
	1.3 to <1.8 overweight	1.8 to <2.3 obesity class I	2.3 to <2.8 obesity class II	2.8–3.3 obesity class III	>3.3 obesity class IV	85th–95th overweight	100–120% 95th obesity class I	>120–140% 95th obesity class II	>140–160% 95th obesity class III	>160% 95th obesity class IV
Females < 10 y	798	2348	2712	1389	395	429	3906	2501	651	155
Age [years]	8.8 (8.0–9.4)	8.8 (7.8–9.4)	8.6 (7.6–9.3)	8.1 (6.8–9.1)	6.7 (5.7–8.1)	8.9 (8.0–9.4)	8.6 (7.5–9.3)	8.5 (7.2–9.3)	8.5 (7.3–9.3)	8.4 (7.1–9.2)
BMI [kg/m^2^]	21.0 (20.2–21.7)	23.1 (21.9–24.0)	25.6 (24.0–26.8)	28.5 (25.7–30.4)	30.2 (27.3–34.2)	20.7 (19.9–21.3)	23.1 (21.9–24.3)	26.6 (25.3–27.9)	30.8 (28.9–32.0)	35.5 (33.5–37.2)
Males < 12 y	1207	4134	5476	2480	550	638	5877	5146	1707	479
Age [years]	10.7 (9.6–11.4)	10.6 (9.5–11.3)	10.3 (9.0–11.2)	9.4 (8.0–10.7)	6.8 (5.8–8.1)	10.7 (9.4–11.3)	10.3 (9.1–11.2)	10.1 (8.7–11.1)	9.9 (8.5–11.1)	10.0 (8.1–11.1)
BMI [kg/m^2^]	22.6 (21.5–23.4)	25.0 (23.7–26.1)	28.1 (26.0–29.8)	31.3 (27.7–34.2)	30.5 (27.8–35.3)	22.0 (21.1–22.7)	24.7 (23.3–26.0)	28.4 (26.6–29.9)	32.6 (30.3–34.1)	37.7 (34.9–40.3)
Females 10 to <14 y	1876	5612	6843	3595	871	1019	8243	6555	2251	729
Age [years]	12.0 (11.0–13.0)	12.0 (11.0–13.0)	12.2 (11.2–13.1)	12.6 (11.6–13.3)	13.0 (12.2–13.6)	12.0 (11.0–13.0)	12.1 (11.1–13.0)	12.3 (11.3–13.2)	12.5 (11.6–13.3)	12.7 (11.7–13.4)
BMI [kg/m^2^]	24.0 (23.0–24.8)	26.6 (25.6–27.6)	30.1 (29.0–31.3)	34.7 (33.6–36.3)	41.3 (40.0–44.0)	23.4 (22.6–24.2)	26.8 (25.6–28.2)	31.2 (29.9–32.6)	36.1 (34.9–37.6)	42.1 (40.4–44.6)
Males 12 to <16 y	1751	5539	7242	3403	591	958	7322	6697	2558	991
Age [years]	13.3 (12.7–14.1)	13.4 (12.7–14.2)	13.6 (12.8–14.5)	14.0 (13.1–15.0)	15.0 (14.0–15.5)	13.4 (12.7–14.1)	13.5 (12.7–14.3)	13.7 (12.9–14.6)	13.8 (12.9–14.8)	14.2 (13.1–15.1)
BMI [kg/m^2^]	24.9 (24.3–25.6)	28.0 (27.0–28.9)	32.0 (30.8–33.4)	37.4 (36.1–39.4)	46.2 (44.3–49.0)	24.4 (23.8–25.0)	28.0 (26.8–29.2)	32.5 (31.2–33.9)	37.4 (36.2–38.8)	43.9 (42.1–46.8)
Females ≥ 14 y	956	2866	4424	4038	2258	605	4983	5241	2506	1266
Age [years]	15.4 (14.7–16.5)	15.3 (14.5–16.4)	15.3 (14.5–16.4)	15.5 (14.6–16.6)	15.7 (14.8–16.6)	15.7 (14.8–16.9)	15.3 (14.5–16.3)	15.3 (14.6–16.4)	15.6 (14.7–16.7)	15.9 (14.9–17.0)
BMI [kg/m^2^]	26.4 (25.7–27.0)	29.0 (28.2–29.8)	32.3 (31.2–33.4)	36.6 (35.2–38.1)	43.1 (40.9–46.5)	25.8 (25.4–26.5)	29.7 (28.4–30.8)	34.3 (33.0–35.7)	39.6 (38.1–41.1)	46.3 (44.1–49.8)
Males ≥ 16 y	100	382	853	1051	854	66	695	1120	750	613
Age [years]	16.9 (16.3–17.4)	16.7 (16.3–17.5)	16.7 (16.3–17.3)	16.9 (16.4–17.5)	17.2 (16.6–18.0)	16.9 (16.3–17.6)	16.7 (16.3–17.5)	16.8 (16.3–17.5)	16.9 (16.4–17.6)	17.1 (16.6–18.0)
BMI [kg/m^2^]	26.9 (26.5–27.4)	29.9 (29.0–30.5)	33.4 (32.3–34.4)	38.1 (36.7–39.8)	46.3 (43.6–50.3)	26.6 (26.0–26.8)	30.7 (29.4–31.7)	35.3 (33.9–36.7)	40.6 (39.2–42.0)	48.2 (45.8–52.4)

Data presented as median and lower/upper quartile range in parentheses.

### CVRFs in females < 10 years

BMI-SDS was related significantly (*p* < 0.001) to systolic blood pressure (β-coefficient 4.0 ± 0.3) and diastolic blood pressure (β-coefficient 2.2 ± 0.3). Systolic and diastolic blood pressures were significantly higher in class IV compared to class III obesity based on the 95th percentile BMI definition, while systolic and diastolic blood pressure did not differ significantly according to the IOTF definition.

Triglycerides, HDL cholesterol, and T/H correlated significantly (*p* < 0.001) with BMI-SDS (β-coefficients 10.1 ± 2.1, −3.6 ± 0.4, 0.4 ± 0.1, respectively), but not LDL or non-HDL cholesterol. None of the lipids differed significantly between classes IV and III obesity regardless of the definition.

AST, ALT, and yGT correlated significantly (*p* < 0.005) with BMI-SDS (β-coefficients 1.1 ± 0.4, 5.7 ± 0.7, 2.5 ± 0.4, respectively). None of the liver enzymes differed significantly between classes IV and III obesity regardless of the definition.

HbA1c and fasting glucose were not related to BMI-SDS, while 2-h glucose in oGTT was related (*p* = 0.001) to BMI-SDS (β-coefficient 3.7 ± 1.1). HbA1c, fasting glucose, and 2-h glucose in oGTT did not differ significantly between classes III and IV obesity regardless of the definition.

### CVRFs in males < 12 years

BMI-SDS was related significantly (*p* < 0.001) to systolic blood pressure (β-coefficient 4.5 ± 0.3) and diastolic blood pressure (β-coefficient 2.6 ± 0.2). Systolic and diastolic blood pressures were significantly higher in class IV compared to class III obesity based on the 95th percentile BMI definition, while systolic and diastolic blood pressure did not differ significantly according to the IOTF definition.

Triglycerides, HDL cholesterol, and T/H correlated significantly (*p* < 0.001) with BMI-SDS (β-coefficients 8.5 ± 1.5, −4.8 ± 0.3, 0.4 ± 0.0, respectively), but not LDL or non-HDL cholesterol. HDL-cholesterol levels were significantly lower and T/H were significantly higher in class IV compared to class III obesity based on the 95th percentile BMI definition but not on the IOTF definition, while none of the other lipids differed significantly between classes IV and III regardless of the definition applied.

AST, ALT, and γGT correlated significantly (*p* < 0.001) with BMI-SDS (β-coefficients 3.5 ± 0.4, 8.7 ± 0.8, 3.4 ± 0.5, respectively). All liver enzymes were significantly higher in class IV compared to class III obesity based on the 95th percentile BMI definition, while there were no significant differences based on the IOTF definition.

Fasting glucose and HbA1 were not related to BMI-SDS, while 2-h glucose in oGTT was related significantly to BMI-SDS (β-coefficient 3.2 ± 1.1). However, their values did not differ significantly between classes III and IV obesity regardless of the definition.

### CVRFs in females 10 to <14 years

BMI-SDS was related significantly (*p* < 0.001) to systolic blood pressure (β-coefficient 8.4 ± 0.2) and diastolic blood pressure (β-coefficient 4.7 ± 0.2). Systolic and diastolic blood pressure were significantly higher in class IV compared to class III obesity regardless of the definition.

Triglycerides, HDL cholesterol, LDL cholesterol, and T/H correlated significantly (*p* = 0.002) with BMI-SDS (β-coefficients 5.6 ± 1.3, −2.0 ± 0.6, −4.5 ± 0.2, 0.3 ± 0.0, respectively), but not non-HDL cholesterol. All lipids except LDL- and non-HDL cholesterol showed statistically significant differences between classes III and IV obesity according to the IOTF definition. Using the 95th percentile BMI definition, only HDL cholesterol and T/H ratio differed significantly between classes III and IV obesity.

AST, ALT, and γGT correlated significantly (*p* < 0.001) with BMI-SDS (β-coefficients 2.1 ± 0.3, 6.9 ± 0.5, 3.9 ± 0.4, respectively). The liver enzyme γGT was significantly higher in class IV compared to class III obesity, while the transaminases did not differ significantly between classes IV and III obesity regardless of the definition.

Fasting glucose and 2-h glucose in oGTT were significantly related to BMI-SDS (β-coefficient 1.2 ± 0.5 and 3.9 ± 0.8, respectively). HbA1c showed no association with BMI-SDS. Both glucose parameters as well as HbA1c did not differ significantly between classes IV and III obesity regardless of the definition.

### CVRFs in males 12 to <16 years

BMI-SDS was related significantly (*p* < 0.001) to systolic blood pressure (β-coefficient 9.7 ± 0.2) and diastolic blood pressure (β-coefficient 5.3 ± 0.2). Systolic and diastolic blood pressure levels were significantly higher in class IV compared to class III obesity regardless of the definition used.

Triglycerides, LDL, HDL, and non-HDL cholesterol, as well as T/H, correlated significantly (*p* < 0.001) with BMI-SDS (β-coefficients 13.2 ± 1.4, 3.2 ± 0.7, −5.6 ± 0.3, 4.3 ± 0.9, 0.6 ± 0.0, respectively). All lipids except LDL cholesterol showed statistically significant differences between classes III and IV obesity regardless of the definition.

AST, ALT, and γGT correlated significantly (*p* < 0.001) with BMI-SDS (β-coefficients 6.1 ± 0.4, 15.0 ± 0.8, 7.2 ± 0.4, respectively). Levels of γGT were significantly higher in class IV compared to class III obesity, while AST did not differ significantly between classes IV and III regardless of the definition. ALT concentrations were significantly higher in class IV obesity compared to class III only if the 95th BMI percentile definition was applied.

Fasting glucose, 2-h glucose in oGTT, and HbA1 correlated significantly (*p* < 0.05) with BMI-SDS (β-coefficients 1.3 ± 0.4, 2.1 ± 0.8, 0.08 ± 0.03, respectively). Their values did not differ significantly between classes III and IV obesity regardless of the definition.

### CVRFs in females > 14 years

BMI-SDS was related significantly (*p* < 0.001) to systolic blood pressure (β-coefficient 6.3 ± 0.2) and diastolic blood pressure (b-coefficient 3.6 ± 0.2). Systolic and diastolic blood pressure values were significantly higher in class IV compared to class III obesity regardless of the definition used.

All lipid parameters were significantly (*p* < 0.001) related to degree of overweight (β-coefficients triglycerides: 12.3 ± 1.1, LDL cholesterol: 2.5 ± 0.6, HDL cholesterol: −4.8 ± 0.2, non-HDL cholesterol: 4.3 ± 0.7, T/H ratio: 0.5 ± 0.0, respectively). All lipids except LDL cholesterol and non-HDL cholesterol showed statistically significant differences between classes III and IV obesity regardless of the definition.

AST, ALT, and γGT correlated significantly (*p* < 0.001) with BMI-SDS (β-coefficients 4.6 ± 0.3, 9.9 ± 0.6, 5.7 ± 0.4, respectively). All liver enzymes were significantly higher in class IV compared to class III obesity regardless of the definition.

Fasting glucose and 2-h glucose in oGTT were significantly related to BMI-SDS (β-coefficient 2.9 ± 0.5 and 3.2 ± 0.7, respectively), but only fasting glucose was significantly higher in class IV compared to class III obesity regardless of the definition. HbA1c showed no association with BMI-SDS.

### CVRFs in males > 16 years

BMI-SDS was related significantly (*p* < 0.001) to systolic blood pressure (β-coefficient 7.4 ± 0.5) and diastolic blood pressure (β-coefficient 5.6 ± 0.4). Systolic and diastolic blood pressure levels were significantly higher in class IV compared to class III obesity using the IOTF definition, in the 95th percentile BMI definition only diastolic blood pressure was significantly higher.

All lipid parameters were significantly related to degree of overweight (β-coefficients triglycerides: 19.4 ± 2.5, LDL cholesterol: 7.3 ± 1.3, HDL cholesterol: −3.6 ± 0.4, non-HDL cholesterol: 8.3 ± 1.6, T/H ratio: 0.7 ± 0.1, respectively). Triglycerides, HDL cholesterol, and the T/H ratio showed statistically significant differences between classes III and IV obesity under both classifications, while LDL and non-HDL cholesterol differed significantly only using the IOTF definition.

AST, ALT, and γGT correlated significantly (*p* < 0.001) with BMI-SDS (β-coefficients 7.1 ± 1.1, 18.3 ± 1.8, 7.1 ± 1.3, respectively). All liver enzymes differed significantly between classes III and IV obesity using the IOTF definition, while using the 95th percentile BMI definition no significant differences were revealed.

Fasting glucose, 2-h glucose in oGTT, and HbA1 were not related to BMI-SDS. Only fasting glucose was significantly higher in class IV compared with class III using the IOTF definition.

### Prevalences of hypertension, dyslipidemia, and T2DM

**Fig. 5:**
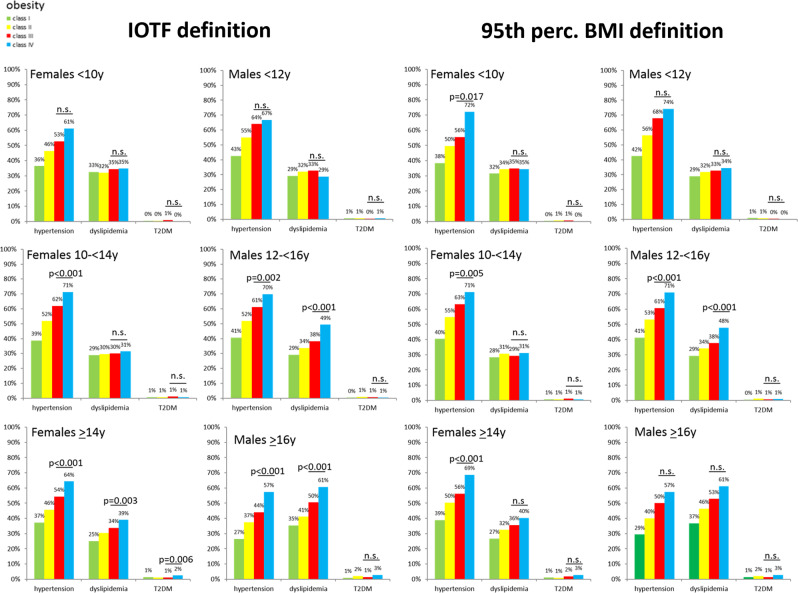
Prevalences of type 2 diabetes mellitus (T2DM), dyslipidemia, and hypertension stratified by age group, sex, and obesity class using two different obesity class definitions.

The prevalences of hypertension, dyslipidemia, and T2DM are given in Fig. 5 stratified by age, sex, and obesity classes. Hypertension was highly prevalent and significantly more frequent in class IV compared to class III obesity regardless of the applied definition in boys 12 to <16 years and girls ≥ 10 years. In girls < 10 years, prevalence of hypertension was significantly higher in class IV compared to class III obesity only using the 95th percentile BMI definition. In boys ≥ 16 years, prevalence of hypertension was significantly higher in class IV compared to class III obesity only when the IOTF definition was used.

Prevalence of dyslipidemia was also considerable but did not differ significantly between classes III and IV obesity in girls < 14 years and boys < 12 years independent of the definition used.

Prevalence of T2DM was generally very low and did not differ significantly between children and adolescents with class IV compared to class III obesity regardless of the definition, age, or sex except females ≥ 14 years. Those girls showed a higher prevalence of T2DM in class IV compared to class III obesity but only when the IOTF definition was used.

### Longitudinal analyses

A total of 1089 children and adolescents with bariatric surgery or with long-term inpatient treatment > 6 months were excluded from the longitudinal analyses. Total number of children included and their respective BMI-SDS with available follow-up are presented in Supplementary Table 1.

Due to the too small percentages of patients with available follow-up (maximum 34% and mean < 20% at 1 year, maximum 19% and mean < 12% at 2 years), we did not analyze the impact of different obesity classes on CVRF longitudinally. The numbers of children with available follow-up decreased with observation period and age at baseline in all six strata.

BMI-SDS reduced significantly (<0.001) in follow-up at 1 and 2 years. The BMI-SDS reduction was not significant different between classes III and IV obesity regardless of the underlying definition, age, or sex except the two subgroups females < 10 years and boys < 12 years: these two groups showed a significantly (*p* < 0.05, respectively) larger decrease in BMI-SDS over 1 and 2 years in the class IV compared to class III obesity according to the IOTF definition but not according to the 95th percentile definition.

## Discussion

To the best of our knowledge, this is the largest study of children and adolescents with classes III and IV obesity presenting at tertiary obesity treatment centers in Central Europe. In line with our hypothesis, many CVRFs did not differ significantly between children with classes III and IV obesity, while there were significant differences in some CVRFs between class III and IV obesity in adolescents.

The relationship between most analyzed CVRFs and classes of obesity was very similar in the IOTF and percentage of 95th percentile of BMI definition. However, in children more significant differences between classes III and IV obesity in respect to CVRFs were found using the 95th BMI percentile definition, while in adolescents the opposite was observed. However, the pattern of significant differences between classes IV and III obesity is difficult to interpret in favor of one of the two classifications, since statistical significance is dependent on effect size and sample size. In the IOTF definition more children and adolescents are classified as class IV or III obese than in the definition based on the percentage of 95th percentile of BMI. This is also reflected in higher BMI values.

We found a linear relationship between degree of overweight expressed as BMI-SDS and systolic and diastolic blood pressure (positive), triglycerides (positive), HDL cholesterol (negative), liver enzymes (positive), and the T/H ratio (positive) as indirect parameter of insulin resistance at any age and sex group. Fasting glucose and 2-h glucose in oGTT correlated significantly to BMI-SDS in some subgroups (e.g., females > 14 years and males 12–16 years). These findings are in line with the study of Skinner et al. highlighting the increased metabolic risk of rising degrees of obesity in childhood [4]. Of interest all these CVRFs are related to insulin resistance [5–8].

However, many of these associations got lost when comparing obesity classes III and IV in different age groups. In prepubertal children (females < 10 years and males < 12 years), only blood pressure levels in both sexes and HDL cholesterol concentrations in boys differed significantly between classes IV and III obesity. Furthermore, this was only true using the 95th percentile BMI definition but not if applying the IOTF definition. Additionally, all other CVRFs did not differ significantly in those children. In pubertal and post pubertal age (females > 10 years and males ≥ 12 years), most CVRFs differed significantly between classes III and IV obesity. However, glucose parameters in any age and sex group (except fasting glucose in females > 14 years) and transaminases in females aged 10–14 years did not differ significantly. Females ≥ 10 years and males ≥ 12 to <16 years with class IV obesity had higher prevalences of hypertension than adolescents with class III obesity independent of the applied obesity class definition, while prevalence of dyslipidemia did not differ significantly between classes IV and III obesity except females ≥ 14 years (only if using the IOTF definition). In males ≥ 16 years, a significant higher prevalence of hypertension was only observed applying the IOTF definition. Independent of age, sex, and used obesity class definition, the prevalences of T2DM did not differ between classes III and IV obesity (except females ≥ 14 years). These findings are in concordance with the hypothesis of Zabarsky et al. questioning a linear relationship between CVRFs and degree of overweight in the upper BMI cut-offs [10]. Besides age and sex, CVRFs are dependent on additional factors such as genetic background, physical fitness, or dietary components apart from degree of overweight. Genetic correlations between childhood obesity versus adult BMI and fasting glucose or T2DM have been shown to be lower, thus supporting the notion that genetic factors contributing to a high BMI in childhood do not substantially overlap with those for the respective parameters of glucose metabolism [22]. Potentially, this low overlap partially explains our nonsignificant correlations between obesity and parameters of glucose metabolism.

Categorization of degrees of obesity according to BMI thresholds is an easy tool. However, the important limitation of this approach is that other important factors influencing CVRFs such as, for example, body fat distribution are neglected [23]. Furthermore, it has been shown in large cohort of Israeli adolescents that also a BMI above the 50th but below the 85th percentile at age of 17 years was associated with cardiovascular morbidity and mortality in the next 40 years [3] demonstrating the limitation of the use of BMI cut-offs. Our analysis emphasizes that categorization by BMI thresholds has shortcomings for distribution of glucose metabolism at any age and for all other CVRFs except blood pressure especially in prepubertal children.

Of note, fasting glucose was related to BMI-SDS in females ≥ 14 years and fasting glucose was significantly higher in females ≥ 14 years with class IV compared to females ≥ 14 years with class III obesity, while in males of any age and in females < 14 years no significant association between BMI-SDS and fasting glucose was observed. This finding is in line with the observation of a female predominance in adolescents with T2DM not only in our study (1.3 versus 1.2% T2DM in adolescents ≥ 14 years, but no difference in children < 14 years) but also within previous reports [24]. This suggests that besides a higher insulin resistance in girls compared to boys [25], the combination of obesity and female sex hormones or associated factors may support the development of T2DM or that male sex hormones or associated factors may prevent the development of T2DM, even if the effect seems to be small. Future studies are necessary to prove these hypotheses.

The vast majority of children attending a specialized treatment center of obesity were lost to follow-up thus casting doubt on the achievement of major improvement in their degree of overweight during the follow-up period. Furthermore, although the children and adolescents with follow-up data achieved a significant decrease of their degree of overweight, this decrease was only marginal and likely not relevant for the CVRFs since the decrease of BMI-SDS was <0.2 on average. It has been shown that a decrease of >0.25 BMI-SDS is necessary to improve CVRFs in children and adolescents [26]. This emphasizes the limitations of short-term inpatient or long-term outpatient standard-of-care conservative obesity management. We found no difference between classes III and IV obesity concerning change of BMI-SDS in females ≥ 10 years and boys > 12 years but a larger degree of BMI-SDS reduction in younger children with class IV obesity compared to class III obesity. This finding is in line with previous studies reporting that especially adolescents with class III or IV obesity in contrast to children with class III or IV obesity do not respond to lifestyle interventions [27, 28].

Almost 10 years ago, we reported that the vast majority of specialized treatment centers for children and adolescents with obesity participating in the APV quality control program were not able to prove the effectiveness of their lifestyle interventions under real-life conditions due to the high drop-out rate of >93% after 2 years [29]. Compared to these historical data, the drop-out rate in our recent analyses decreased probably due to the APV benchmarking process improving the quality of interventions. However, the drop-out rate is still too high to prove effectiveness. This finding highlights that achieving treatment adherence is the greatest challenge in treating children and adolescents with obesity. The causes of nonadherence have to be analyzed in order to judge if it is based on characteristics of patients with obesity (e.g., difficulties to visit the treatment centers regularly) or the interventions itself (e.g., not tailored specific underfinanced intervention with insufficient patient contacts). The problem of nonadherence has to be solved before the analysis of effectiveness of treatment strategies such as bariatric surgery, medications, or more intensified lifestyle interventions.

The strengths of our study are the very large study sample, the multicenter design, and the analysis under real-life conditions and not under ideal circumstances of study trials with selected patients. However, we have to mention some important limitations of the study. First, not all obesity treatment centers in the analyzed region participated in this study. Nevertheless, the most specialized treatment centers are likely to be enrolled in this study at least in Germany, since participating in the APV quality program is necessary for funding of intervention by German health insurances and accreditation of obesity treatment centers. Second, our reference group of subjects with overweight or obesity was referred to specialized obesity clinics. Therefore, they may be characterized by a more pronounces CVRF profile compared to subjects with similar anthropometric measures derived from the population. Third, we do not have complete data on pubertal stages. Therefore, the three age categories used in this study are only an approximation of pubertal stage. Fourth, we performed no study visits to monitor for correct measurement, for example, of blood pressure. However, data were determined by the obesity specialist centers according to German guidelines. Finally, the drop-out rate at follow-up was too high to calculate longitudinal relationships between CVRFs and changes of weight status.

In summary, we demonstrated the great challenge of treatment adherence in children and adolescents with obesity. We showed that prevalences of hypertension and dyslipidemia did not differ significantly or consistently between classes III and IV obesity in males ≤ 12 years and females ≤ 10 years. Disturbances of glucose metabolism including T2DM did not differ significantly between classes III and IV obesity in boys of any age or in girls < 14 years. Therefore, the presence of CVRFs and not the BMI cut-offs should primarily guide treatment decisions such as medications or bariatric surgery. More longitudinal studies with high follow-up rate are needed to justify decisions being based solely on obesity grades.

## Supplementary information

supplemental Table 1
