# Supplementary material for: Comparison of cardiovascular risk factors between children and adolescents with classes III and IV obesity: findings from the APV cohort
Source: Int J Obes (Lond). 2021 Apr 7;45(5):1061–73. doi: 10.1038/s41366-021-00773-x (PMC8081660; doi:10.1038/s41366-021-00773-x)
Supplement: Supplementary file 1 — supplemental Table 1 [file 41366_2021_773_MOESM1_ESM.docx]

Table 1 Supplement: Number of patients and percentage of entire cohort compared to baseline with available follow-up and their change of BMI-SDS stratified by age-group, sex, and obesity class using two different obesity class definitions

|  |  | BMI- | SDS |  |  |  | BMI | percentile |  |  |
| --- | --- | --- | --- | --- | --- | --- | --- | --- | --- | --- |
|  | 1.3-<1.8  overweight | 1.8-<2.3  obesity  class I | 2.3-<2.8  obesity  class II | 2.8-3.3  obesity  class III | >3.3  obesity  class IV | 85-95th  overweight | 100%-120% 95th  obesity  class I | 120%-140% 95th  obesity  class II | >140-160% 95th  obesity  class III | >160% 95th  obesity  class IV |
| Females  <10y | 798 | 2348 | 2711 | 1389 | 395 | 429 | 3906 | 2500 | 651 | 155 |
| 1y follow-up  ΔBMI-SDS | 236 (29.6%)  -0.1 (-0.3-0.1) | 795 (33.9%)  -0.2 (-0.4-0.0) | 826 (30.5%)  -0.2 (-0.4-0.0) | 391 (28.1%)  -0.2 (-0.4- -0.1) | 102 (25.8%)  -0.3 (-0.4- -0.2) | 124 (28.9%)  -0.1 (-0.3-0.1) | 1258 (32.2%)  -0.2 (-0.4-0.0) | 741 (29.6%)  -0.2 (-0.4-0.0) | 185 (28.4%)  -0.2 (-0.4- -0.1) | 42 (27.1%)  -0.2 (-0.4- -0.1) |
| 2y follow-up  ΔBMI-SDS | 140 (17.5%)  0.0 (-0.2-0.2) | 437 (18.6%)  -0.1 (-0.3-0.1) | 469 (17.3%)  -0.2 (-0.4-0) | 214 (15.4%)  -0.2 (-0.4- -0.1) | 51 (12.9%)  -0.3 (-0.7- -0.2) | 81 (18.9%)  0.0 (-0.1-0.3) | 689 (17.6%)  -0.1 (-0.4-0.1) | 431 (17.2%)  -0.2 (-0.4-0.0) | 89 (13.7%)  -0.3 (-0.5- -0.1) | 21 (13.5%)  -0.3 (-0.7- -0.2) |
| Males  <12y | 1207 | 4134 | 5475 | 2478 | 549 | 638 | 5877 | 5145 | 1706 | 477 |
| 1y follow-up  ΔBMI-SDS | 308 (25.5%)  -0.1 (-0.4-0.1) | 1198 (29.0%)  -0.2 (-0.4-0.0) | 1515 (27.7%)  -0.2 (-0.3-0.0) | 670 (27.0%)  -0.2 (-0.3- -0.1) | 125 (22.8%)  -0.3 (-0.4- -0.1) | 136 (21.3%)  -0.1 (-0.4-0.1) | 1701 (28.9%)  -0.2 (-0.4-0.0) | 1439 (28.0%)  -0.2 (-0.3- -0.1) | 421 (24.7%)  -0.2 (-0.3- -0.1) | 119 (24.9%)  -0.2 (-0.3- -0.1) |
| 2y follow-up  ΔBMI-SDS | 176 (14.6%)  -0.1 (-0.4-0.2) | 662 (16.0%)  -0.2 (-0.4-0.1) | 795 (14.5%)  -0.2 (-0.4-0.0) | 343 (13.8%)  -0.2 (-0.4- -0.1) | 79 (14.4%)  -0.4 (-0.7- -0.2) | 83 (13.0%)  -0.1 (-0.4-0.2) | 958 (16.3%)  -0.2 (-0.4-0.1) | 745 (14.5%)  -0.2 (-0.4-0.0) | 208 (12.2%)  -0.2 (-0.4- -0.1) | 61 (12.8%)  -0.2 (-0.6- -0.1) |
| Females  10-<14y | 1876 | 5608 | 6821 | 3570 | 837 | 1019 | 8237 | 6525 | 2229 | 702 |
| 1y follow-up  ΔBMI-SDS | 422 (22.5%)  -0.2 (-0.4-0.1) | 1440 (25.7%)  -0.2 (-0.4-0.0) | 1541 (22.6%)  -0.1 (-0.3-0.1) | 692 (19.4%)  -0.1 (-0.2-0.1) | 129 (15.4%)  0.0 (-0.2-0.1) | 219 (21.5%)  -0.1 (-0.4-0.1) | 2048 (24.9%)  -0.2 (-0.4-0.0) | 1419 (21.7%)  -0.1 (-0.3-0.1) | 431 (19.3%)  -0.1 (-0.2-0.1) | 107 (15.2%)  0.0 (-0.2-0.1) |
| 2y follow-up  ΔBMI-SDS | 217 (11.6%)  -0.2 (-0.5-0.2) | 744 (13.6%)  -0.1 (-0.5-0.2) | 748 (11.0%)  -0.1 (-0.3-0.2) | 326 (9.1%)  0.0 (-0.3-0.2) | 70 (8.4%)  0.1 (-0.1-0.2) | 117 (11.5%)  -0.2 (-0.5-0.1) | 1060 (12.9%)  -0.1 (-0.4-0.2) | 661 (10.1%)  0.0 (-0.3-0.2) | 204 (9.2%)  0.0 (-0.3-0.2) | 63 (9.0%)  0.1 (-0.1-0.3) |
| Males  12-<16y | 1750 | 5520 | 7194 | 3327 | 519 | 958 | 7297 | 6649 | 2498 | 908 |
| 1y follow-up  ΔBMI-SDS | 287 (16.4%)  -0.1 (-0.4-0.1) | 1094 (19.8%)  -0.2 (-0.4-0.0) | 1269 (17.6%)  -0.1 (-0.3-0.0) | 466 (14.0%)  -0.1 (-0.2-0.1) | 79 (15.2%)  0.0 (-0.2-0.1) | 138 (14.4%)  -0.1 (-0.4-0.1) | 1420 (19.5%)  -0.2 (-0.4-0.0) | 1142 (17.2%)  -0.1 (-0.3-0.1) | 360 (14.4%)  -0.1 (-0.2-0.1) | 135 (14.9%)  0.0 (-0.2-0.1) |
| 2y follow-up  ΔBMI-SDS | 143 (8.2%)  -0.1 (-0.4-0.3) | 542 (9.8%)  -0.1 (-0.4-0.2) | 610 (8.5%)  -0.1 (-0.3-0.2) | 217 (6.5%)  0.0 (-0.3-0.2) | 35 (6.7%)  0.1 (-0.1-0.2) | 69 (7.2%)  -0.2 (-0.5-0.2) | 704 (9.6%)  -0.1 (-0.4-0.2) | 539 (8.1%)  0.0 (-0.3-0.2) | 170 (6.8%)  0.0 (-0.3-0.2) | 65 (7.2%)  0.1 (-0.1-0.3) |
| Females  >14y | 951 | 2836 | 4354 | 3858 | 2032 | 603 | 4935 | 5126 | 2344 | 1082 |
| 1y follow-up  ΔBMI-SDS | 97 (10.2%)  -0.2 (-0.5-0.0) | 358 (12.6%)  -0.2 (-0.5-0.0) | 558 (12.8%)  -0.2 (-0.5-0.0) | 426 (11.0%)  -0.2 (-0.4-0.0) | 246 (12.1%)  -0.2 (-0.4- -0.1) | 49 (8.1%)  -0.2 (-0.5-0.0) | 613 (12.4%)  -0.2 (-0.5-0.0) | 651 (12.7%)  -0.2 (-0.5-0.0) | 245 (10.5%)  -0.2 (-0.4-0.0) | 129 (11.9%)  -0.2 (-0.3- -0.1) |
| 2y follow-up  ΔBMI-SDS | 47 (4.9%)  -0.2 (-0.6-0.1) | 154 (5.4%)  -0.2 (-0.5-0.1) | 238 (5.5%)  -0.2 (-0.6-0.1) | 186 (4.8%)  -0.3 (-0.5-0.0) | 111 (5.5%)  -0.3 (-0.6- -0.1) | 25 (4.1%)  -0.1 (-0.5-0.1) | 264 (5.3%)  -0.2 (-0.5-0.1) | 285 (5.6%)  -0.2 (-0.5-0.0) | 110 (4.7%)  -0.3 (-0.6- -0.1) | 53 (4.9%)  -0.3 (-0.5- -0.1) |
| Males  >16y | 98 | 378 | 827 | 975 | 691 | 63 | 686 | 1063 | 676 | 484 |
| 1y follow-up  ΔBMI-SDS | 8 (8.2%)  -0.3 (-0.8- -0.1) | 43 (11.4%)  -0.3 (-0.6-0.0) | 63 (7.6%)  -0.2 (-0.4-0.0) | 82 (8.4%)  -0.1 (-0.4-0.1) | 69 (10.0%)  -0.2 (-0.4-0.0) | 6 (9.5%)  -0.3 (-0.7- -0.1) | 69 (10.1%)  -0.2 (-0.5-0.0) | 80 (7.5%)  -0.2 (-0.4-0.1) | 54 (8.0%)  -0.1 (-0.4-0.0) | 57 (11.8%)  -0.2 (-0.5-0.0) |
| 2y follow-up  ΔBMI-SDS | N/A | 16 (4.2%)  -0.5 (-0.8-0.0) | 32 (3.9%)  -0.2 (-0.4-0.1) | 34 (3.5%)  -0.1 (-0.4-0.2) | 42 (6.1%)  -0.2 (-0.7-0.1) | N/A | 29 (4.2%)  -0.4 (-0.8-0.0) | 38 (3.6%)  0.0 (-0.2-0.2) | 22 (3.3%)  -0.2 (-0.8-0.0) | 36 (7.4%)  -0.2 (-0.6-0.1) |

Data as percentage or median and lower/upper quartile in parentheses
